# Selective Block of Upregulated Kv1.3 Potassium Channels in ON-Bipolar Cells of the Blind Retina Enhances Optogenetically Restored Signaling

**DOI:** 10.3390/ijms241814207

**Published:** 2023-09-18

**Authors:** Giulia Schilardi, Jakub Kralik, Sonja Kleinlogel

**Affiliations:** Institute of Physiology & Department of Biomedical Research (DBMR), Graduate School for Cellular and Biomedical Sciences (GCB), University of Bern, 3012 Bern, Switzerland; giulia.schilardi@unibe.ch (G.S.); jakub.kralik@roche.com (J.K.)

**Keywords:** ON-bipolar cells, optogenetic gene therapy, Kv1.3 channel, Psora-4, retinal degeneration, vision restoration, Opto-mGluR6, patch clamp, multi-electrode array recordings

## Abstract

Loss of photoreceptors in retinal degenerative diseases also impacts the inner retina: bipolar cell dendrites retract, neurons rewire, and protein expression changes. ON-bipolar cells (OBCs) represent an attractive target for optogenetic vision restoration. However, the above-described maladaptations may negatively impact the quality of restored vision. To investigate this question, we employed human post-mortem retinas and transgenic *rd1_Opto-mGluR6* mice expressing the optogenetic construct Opto-mGluR6 in OBCs and carrying the retinal degeneration *rd1* mutation. We found significant changes in delayed rectifier potassium channel expression in OBCs of degenerative retinas. In particular, we found an increase in Kv1.3 expression already in early stages of degeneration. Immunohistochemistry localized Kv1.3 channels specifically to OBC axons. In whole-cell patch-clamp experiments, OBCs in the degenerated murine retina were less responsive, which could be reversed by application of the specific Kv1.3 antagonist Psora-4. Notably, Kv1.3 block significantly increased the amplitude and kinetics of Opto-mGluR6-mediated light responses in OBCs of the blind retina and increased the signal-to-noise ratio of light-triggered responses in retinal ganglion cells. We propose that reduction in Kv1.3 activity in the degenerated retina, either by pharmacological block or by KCNA3 gene silencing, could improve the quality of restored vision.

## 1. Introduction

Bipolar cells, the first retinal interneurons downstream from the photoreceptors, play a key role in retinal processing. They filter and relay distinct components of the light signal to the inner retina [1,2]. Bipolar cells are divided into ON-bipolar cells (OBCs) and OFF-bipolar cells [3,4]. OBCs are again divided into rod and cone OBCs, depending on their connectivity to rod and cone photoreceptors, respectively. In many degenerative diseases of the retina, such as retinitis pigmentosa (RP) [5,6], the death of photoreceptors disrupts retinal signaling, rendering the retina insensitive to light. RP is a rare genetic disorder with a prevalence of 1/4000, and there are more than 300 genes involved [7]. In RP, rods degenerate, followed by a late degeneration of cones, ultimately resulting in blindness [7,8]. Currently, there is only one FDA-approved gene supplementation therapy available for genetic blindness, for patients specifically carrying the biallelic mutation of the RPE65 gene [9]. Given the heterogenous genetic origin of RP, other, more universal, therapeutic approaches are being explored, amongst them electronic prostheses [10,11], stem cell transplantation [12,13], expression of photo-switchable ligands [14,15], and optogenetic gene therapy [9,16,17]. Optogenetic gene therapy was shown to restore some visual function in a human patient [18] and in murine models of RP, particularly when targeted to the OBCs [19,20,21,22] such as Opto-mGluR6 [20,22].

Despite the successful reintroduction of light sensitivity via OBC-targeted optogenetic gene therapy, functional restoration may be further improved. It is known that retinal degeneration causes maladaptations within the inner retina, such as retraction of bipolar cell dendrites [23,24], aberrant hyperactivity [25], neuronal rewiring [26], and changes in gene and protein expression [23,27,28]. The expression and localization of ion channels in OBCs seem to be affected as well [29,30,31]. By modulating the membrane potential, ion channels are fundamental for the ability of a cell to transmit a signal. In the degenerated retina, big conductance (BK) potassium channels were shown to be downregulated [31]. However, OBCs express other potassium channels with a unique subcellular distribution as well [32,33,34]. For example, three subtypes of delayed rectifier channels from the Kv1 family were immunohistochemically identified in the murine retina already in 1995: Kv1.1, Kv1.2, and Kv1.3 [35].

In the present study, we show that Kv1.3 is only expressed in the degenerating retina with a specific axonal localization, both in the murine rd1 retina and in the post-mortem human retina. This renders OBCs in the degenerated retina less responsive. We show here that a specific pharmacological block of Kv1.3 channels in the blind degenerated retina improves amplitude and kinetics of Opto-mGluR6 triggered light-responses in OBCs, as well as the signal-to-noise ratio of light-responses from RGCs.

## 2. Results

### 2.1. Kv1.3 Is Expressed in OBC Axons of rd1 (FVB/NCrl_Opto-mGluR6) Mouse Retina

**Figure 1:**
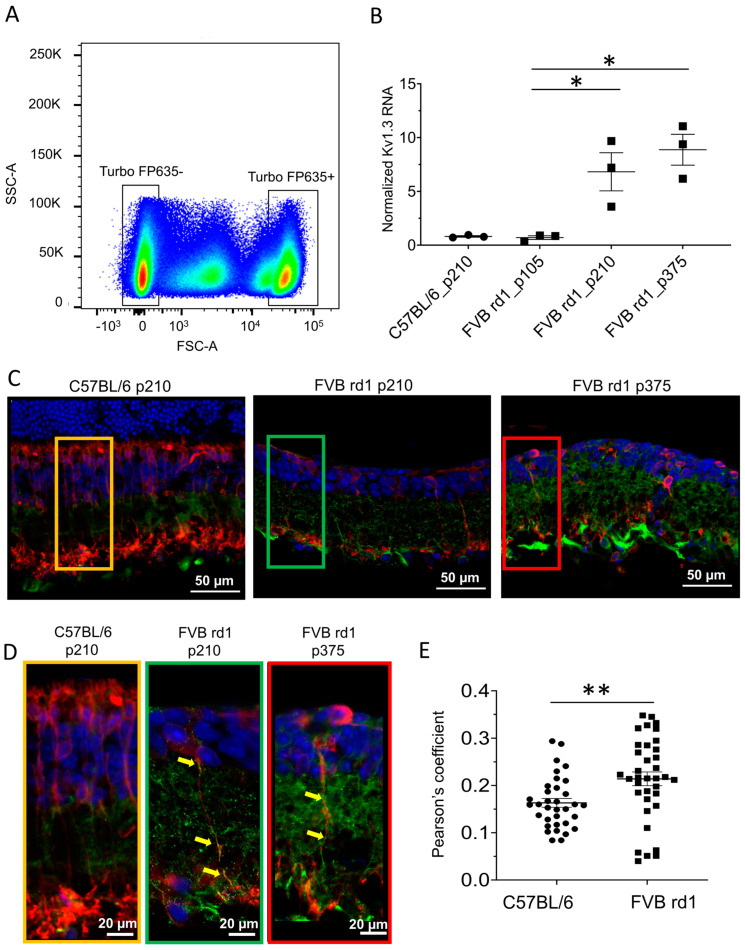
Kv1.3 channel expression in OBCs is triggered by retinal degeneration. (**A**) Illustrative FACS scatter plot of red-fluorescing (TuboFP635) OBCs with positive and negative cell populations from a pool of 10 dissociated retinas; (**B**) Kv1.3 channel RNA levels in OBCs from *C57BL/6* mice (*p* 210), *FVB rd1* mice (p105–p210, * *p* = 0.026 and p375, * *p* = 0.027) normalized to the housekeeping gene RPL8. (**C**) Anti-Kv1.3 immunolabeling (green) on retinal cryosection of *C57BL/6* mice at p210 (**left**), *FVB rd1* mice at p210 (**middle**), and p375 (**right**). Rod-OBCs were identified by anti-PKCα immunolabeling (red), DAPI in blue. Images were taken as single optical sections (770 nm) on a Zeiss LSM880 confocal microscope (40×, NA: 1.3). Co-staining with Gαo is shown in Supplementary Figure S2. (**D**) Magnification of the marked areas in C. Kv1.3 channels (green spots on cell axons) are marked by yellow arrows. (**E**) Pearson’s coefficients for Kv1.3 and PKCα co-localization (** *p* = 0.0036). Data points from C57/BL6 retinas are depicted as circles (●), and data points from FVB rd1 retinas are depicted as squares (▪).

We extracted ON-bipolar cells from retinas of wild-type *C57BL/6J_Opto-mGluR6* (*C57BL/6*) and degenerated *FVB/NCrl_Opto-mGluR6* (*n*) mice expressing the optogenetic protein Opto-mGluR6 linked to the fluorescent reporter TurboFP635 specifically in their OBCs [36] (see Methods for details). TurboFP635 was used for fluorescence-activated ON-bipolar cell sorting (Figure 1A). We subsequently determined Kv1.3 mRNA levels from wild-type *C57BL/6* (positive control, N = 10 retinas, 3 biological samples) and degenerated *FVB rd1* OBC extracts (negative control, N = 10, 3 biological samples). We investigated three time points (p105, p210, p375) in the *FVB rd1* model, representing the three main stages of degeneration [37,38]. Values obtained for Kv1.3 channel RNA were normalized against the RNA levels of the housekeeping gene RPL8, which remains stable during retinal degeneration [31,39]. As shown in Figure 1B, Kv1.3 mRNA is absent in *C57BL/6* (0.81 ± 0.07%) and OBCs from early degeneration *FVB rd1* retinas (p105: 0.70 ± 0.17%), but it increases rapidly and significantly as degeneration progresses (p210: 6.82 ± 1.77%, * *p* = 0.0263; p375: 8.87 ± 1.43%, * *p* = 0.0271). We further investigated mRNA extracts from OBCs of the slower degenerating *C3H/HeOuJ_Opto-mGluR6 rd1 (C3H/HeOuJ rd1)* retina [36] and found the same upregulation of Kv1.3 (p105: 0.61 ± 0.071%; p210: 1.31 ± 0.057%, ** *p* = 0.015) (Supplementary Figure S1), confirming that Kv1.3 upregulation is a common denominator of retinal degeneration and not mouse-line specific.

The massive increase in Kv1.3 mRNA expression in OBCs of the *rd1* retina was also corroborated by immunolabeling of the Kv1.3 protein on retinal cryosections (Supplementary Figure S2). To identify the specific subcellular compartment of Kv1.3 expression, we used the OBC markers anti-Goα (Supplementary Figure S2) and anti-PKCα (Figure 1C,D) [39]. Kv1.3 expression was clearly confined to rod-OBC axons (Figure 1D, Pearson’s coefficients Kv1.3 vs. PKCα Figure 1E, *C5BL/6J*: 0.159 ± 0.027, N = 34 rod-OBCs; *FVB rd1*: 0.214 ± 0.037, N = 33 rod-OBCs, ** *p* = 0.0036).

**Figure 2:**
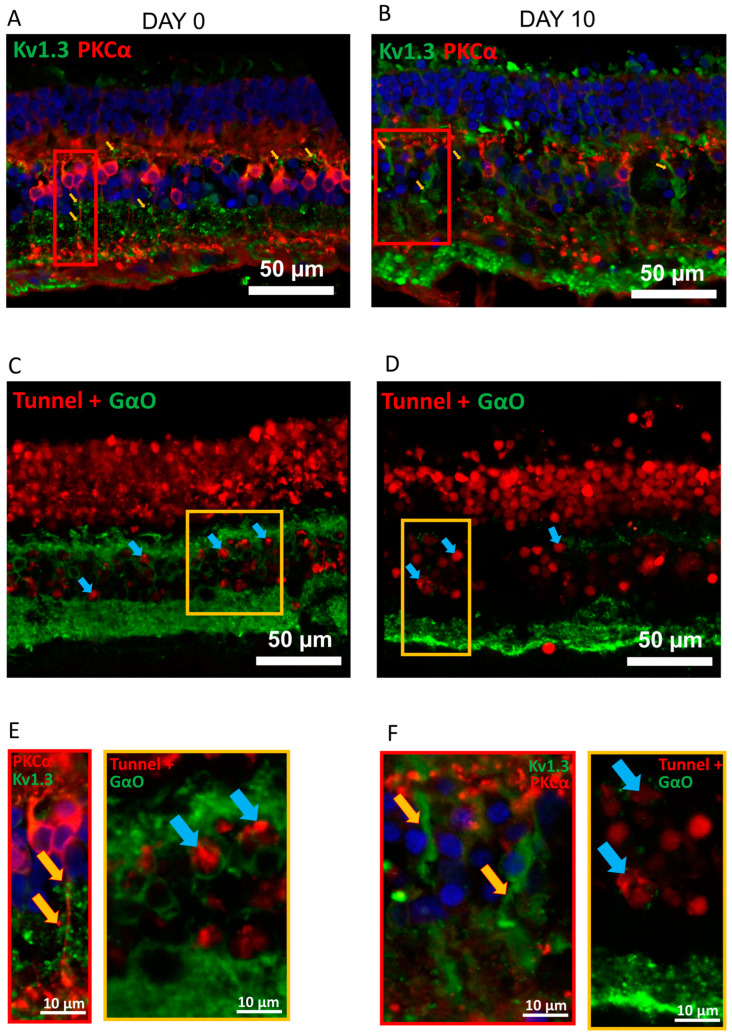
The post-mortem human retina is a degenerative model with Kv1.3 expressed in OBC axons. (**A**) Anti-Kv1.3 immunolabeling (green) of a retinal cryosection from a human donor at day 0 (approximately 24 h post-mortem) and (**B**) at day 10 of culturing. Kv1.3 expression on OBC axons is highlighted with yellow arrows. Rod-OBCs were identified by anti-PKCα immunolabeling (red). (**C**) TUNEL+ (red) and anti-Gαo (green, OBCs) co-staining at 0 days and (**D**) 10 days in culture. Apoptotic cells are highlighted with blue arrows. (**E**) Magnification of figures in (**A**) (red rectangles) and (**C**) (yellow rectangles). (**F**) Magnification of figures in (**B**) (red rectangles) and (**D**) (yellow rectangles). Images were taken as single optical sections (770 nm) on a Zeiss LSM880 confocal microscope (40×, NA: 1.3). Refer to Supplementary Figure S3 for corresponding photomicrographs of donors 2 and 3.

We observed similar expression of Kv1.3 in human post-mortem retinal explants (Figure 2A,B (donor 1), Supplementary Figure S3 donors 2 and 3) 12–24 h post-mortem (Pearson’s coefficient Kv1.3/PKCa co-localization 0.242 ± 0.037, N = 42 OBCs from 3 donor retinas). Kv1.3 is identified as green puncta (Figure 2E, yellow arrows). However, Kv1.3 was not only confined to the axons but was also expressed in OBC dendrites, which are still present in the post-mortem human retina (Figure 2A, yellow arrow). The axonal Kv1.3 antibody signal increased further at 10 days in culture (Figure 2B,F). This indicates that human retinal cultures were already in a degenerative state at day 0 (12–48 h post-mortem; Figure 2 and Figure S3). Since Kv1.3 is reported to be involved in apoptotic pathways [40,41], we additionally stained explant cryosections for TUNEL-positive cells and confirmed apoptosis at day 0 (Figure 2C,E), with about half of OBCs (11 ± 1 out of 23 ± 2 in 27.03 ± 2.15 mm^2^ retina, three donors, three sections each) labeling TUNEL-positive. TUNEL+ cells were also present in the inner nuclear layer at 10 days of culturing (Figure 2D,F). In comparison, no TUNEL+ OBCs were found in retinal slices from healthy wild-type mice (Supplementary Figure S4). These observations suggest that Kv1.3 expression in OBCs may be a common denominator of degeneration in murine and human retina.

### 2.2. The Delayed Rectifier Potassium Current Is Driven by Kv1.3 in OBCs of the Degenerated FVB rd1 Retina

**Figure 3:**
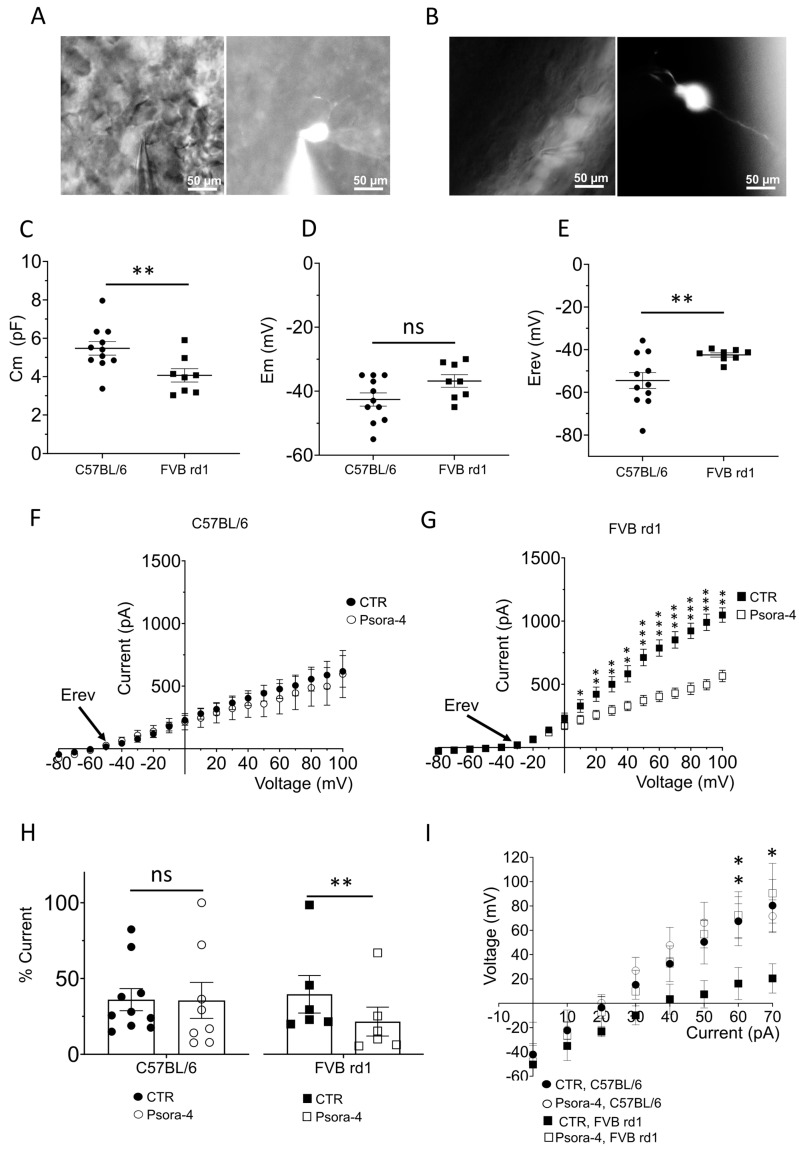
The Kv1.3 channel shapes the potassium current in OBCs of the degenerated retina. Epifluorescent micrographs from (**A**) retinal slices of *C57BL/6* (**left**) showing TurboFP635 expressing OBCs (**right**) and in (**B**) retinal whole mounts of *FVB rd1* mice (**left**) showing OBCs injected with Lucifer Yellow during the electrophysiological recording (**right**); pictures were taken with a Nikon Eclipse E600FN (40×, NA: 0.80) microscope equipped with an infrared GP-CAM3 Altair Astro camera. (**C**–**E**) Average cell membrane capacitances ((**C**), Cm, ** *p* = 0.011), resting membrane potentials ((**D**), Em, *p* = 0.059), and reversal membrane potentials ((**E**), Erev, ** *p* = 0.009) for OBCs of *C57BL/6* (N = 11) and *FVB rd1 6* (N = 8) mice. (**F**,**G**) I/V relationship (step protocol from −80 mV to 100 mV, Δ = 10 mV) in bath (CTR) and during Psora-4 application (100 nM, N = 9) in (**F**) *C57BL/6* retinas (N (Ctrl) = 11, N (Psora) = 9) and in (**G**) *FVB rd1* retinas (N (Ctrl) = 8, N (Psora) = 8; 10 mV, * *p* = 0.0176; 20 mV, ** *p* = 0.0032; 30 mV, ** *p* = 0.0022; 40 mV, ** *p* = 0.0013; 50 mV, *** *p* = 0.0026; 60 mV, *** *p* = 0.0009; 70 mV, *** *p* = 0.0006; 80 mV, *** *p* = 0.0006; 90 mV, *** *p* = 0.0005; 100 mV, ** *p* = 0.0305). (**H**) Comparison of % current at 100 mV in bath solution for C57BL/6 (**left**) (CTR, N = 10) and with 100 nM Psora-4 (N = 8) (*p* = 0.346), and *FVB rd1* (**right**) (CTR, N = 6), 100 nM Psora-4 (N = 6), ** *p* = 0.0031. (**I**) Average OBC membrane potentials in response to current injection steps (from 0 pA to 70 pA, Δ = 10 mV) in *C57BL/6* retinas in control conditions (CTR, N = 6) and with 100 nM Psora-4 (N = 6) and in *FVB rd1* retinas in bath (CTR, N = 7) and with Psora-4 (N = 4), (60 mV) ** *p* = 0.02; (70 mV) * *p* = 0.05. Data points from C57/BL6 retinas are depicted as circles (●), and data points from *FVB rd1* retinas are depicted as squares (▪).

To investigate the physiological consequences of Kv1.3 expression in OBC axons of the degenerated retina, we performed whole-cell patch-clamp recordings from OBCs of *FVB rd1* retinal whole mounts (Figure 3A) and *C57BL/6* retinal slices (Figure 3B). The lack of photoreceptors in *FVB rd1* retinas allowed direct access to the OBCs from the photoreceptor side of the whole mount [22,31,42], whereas vertical vibratome sections were cut from *C57BL/6* retinas. OBCs were targeted by TurboFP635 fluorescence (Figure 3A,B; left) and patched cells labeled by 0.03% Lucifer yellow contained in the intracellular solution for subsequent identification [43,44] (Figure 3A,B; right). OBCs from *FVB rd1* retinas had a significantly decreased membrane capacitance (Cm, pF) (4.06 pF ± 0.34, N = 8) compared to OBCs in *C57BL/6* retinas (5.37 pF ± 0.35, N = 11; ** *p* = 0.011; Figure 3C), indicative of the reduced cell surface area as a consequence of dendrite loss during the degenerative process [23,31]. Membrane potentials were not significantly different between the two lines (Figure 3D, *p* = 0.059; *C57BL/6*, −42.64 mV ± 2.07, N = 11; *FVB rd1* −36.84 mV ± 1.9, N = 8); however, the reversal potential during ramp stimuli of OBCs in *FVB rd1* retinas was significantly depolarized (−42.51 mV ± 0.99, N = 8) compared to *C57BL/6* retinas (−54.52 mV ± 3.71, N = 11; ** *p* = 0.009; Figure 3E–G), suggesting a modification in outward current composition. For this reason, we additionally stained retinal slices for Kv1.1 and Kv1.2 channels, which were documented to be expressed in retinal OBCs [35]. Kv1.2 was localized to the OBC dendrites and axon terminals, while Kv1.1 was confined to the OBC dendrites (Supplementary Figure S5A,B), indicative of a function in synaptic integration. Accordingly, the unspecific potassium channel antagonist TEA (tetraethylammonium, 10 mM) reduced the outward current of *C57BL/6* OBCs significantly (60 mV, ** *p* = 0.008, N = 3; Supplementary Figure S5C), whereas the specific Kv1.3 channel blocker Psora-4 had no effect (Figure 3F). Conversely, in the degenerated *FVB rd1* retina where OBC dendrites are lacking, Kv1.1. and Kv1.2 channels are no longer expressed (Supplementary Figure S6). Instead, the Kv.1.3 channel is expressed, as confirmed by a significant current decrease during specific antagonist (Psora-4) application (Figure 3G). Kv1.3 expression was corroborated by the % current (Itot) at 100 mV, which was reduced to 21.90 ± 9.69% (CTR: 40.23 ± 12.58%, ** *p* = 0.0031, N = 6, Figure 3H) when Psora-4 was applied, but this occurred only in *FVB rd1* OBCs and not in *C57BL/6* OBCs (CTR: 43.66 ± 7.29%, Psora-4: 38.51 ± 13.68%, N = 8; *p* = 0.346; Figure 3H). % current was calculated by normalization to the total current at 100 mV. Also, the kinetic features of the Kv currents differed in OBCs of the *C57BL/6* and the *FVB rd1 retina* (Supplementary Figure S7). Kv channels in the healthy retina showed virtually no inactivation (Tau(off) = 564.37 ± 147.72 ms), whereas the currents rapidly inactivated in the degenerating *FVB rd1* retina (43.42 ± 22.43 ms), indicative of Kv1.1 [45] and Kv1.3 channels [46], respectively. Together, these results show that Kv1.1 and Kv1.2 dominate the potassium current in OBCs of the healthy retina, whereas it is Kv1.3 that dominates in OBCs of the degenerated retina.

Since regulation of the membrane potential is fundamental for the propagation of a light-activated signal in OBCs, we evaluated the effect of Kv1.3 inhibition on the cell membrane potential (Figure 3I). OBCs of *FVB rd1* retinas at p210 showed a reduced capacity to respond to the current application compared to the healthy *C57BL/6* retina (60 mV, ** *p* = 0.02, 70 mV, * *p* = 0.05), particularly at depolarized membrane potentials (Figure 3I) [31]. Psora-4 application reversed this disadvantageous effect entirely, allowing OBCs to regain responsiveness, suggesting that a specific block of Kv1.3 channels may allow OBCs to regain some of their healthy physiological properties.

### 2.3. Kv1.3 Inhibition Accelerates and Enhances the Light-Induced, Opto-mGluR6-Mediated Conductance in OBCs of the Degenerated Retina

**Figure 4:**
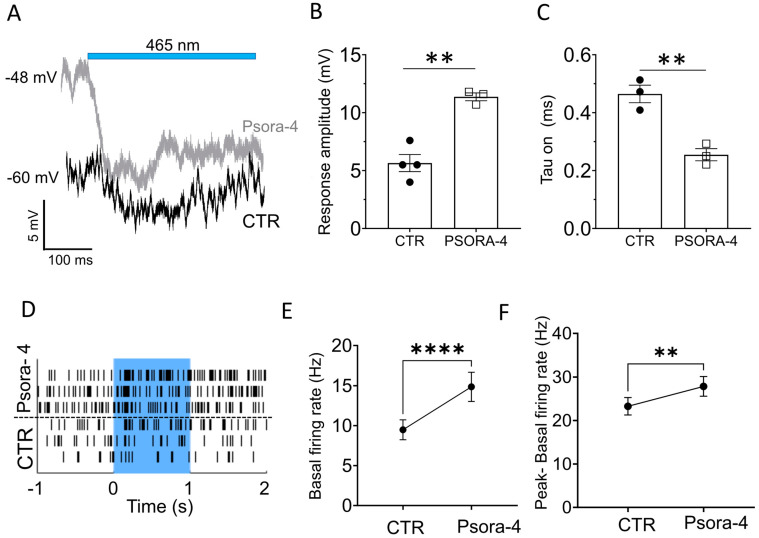
The specific Kv1.3 antagonist Psora-4 enhances light responses in the degenerated and optogenetically treated mouse retina. (**A**) Exemplar traces of *FVB rd1* OBC (p210) responses to light in control conditions (black) and during application of Psora-4 (gray). For a detailed explanation of the membrane potential tuning, see Supplementary Figure S8. (**B**) Light response amplitudes of *FVB rd1* OBCs (p210) in control conditions (CTR, N = 4) and with Psora-4 (N = 3), ** *p*= 0.0019. (**C**) Tau(on) values of the light responses presented in (**B**) in control conditions (CTR) and with Psora-4, ** *p* = 0.006. Data points for control conditions are depicted as circles (●), and data points for Psora-4 are depicted as empty squares (▪). (**D**) Exemplar MEA single electrode raster plots from an *FVB rd1* retina for 3 consecutive light stimulations (blue underlay) with and without bath application of Psora-4. (**E**,**F**) Psora-4 application in *FVB rd1* retinas resulted in a significant increase in the spontaneous firing rate ((**E**), **** *p* = <0.0001, N = 69 electrodes) and a significant increase in light responses ((**F**), ** *p* = 0.002). Light stimulus: 1 s, 465 nm, 5 × 10^15^ photons/cm^2^/s.

Since the blockade of Kv1.3 channels re-establishes not only cell responsiveness but also membrane potential regulation and repolarization in OBCs of the degenerated retina, we hypothesized that Kv1.3 antagonism may improve optogenetically elicited responses by Opto-mGluR6 in OBCs. We performed perforated-patch clamp recordings from OBCs of *FVB rd1* retinal whole mounts at *p210* (Figure 4A). Light stimulation (1 s, 465 nm, 5 × 10^15^ photons/cm^2^/s) of Opto-mGluR6 resulted in characteristic OBC hyperpolarizations [20,22] in both control conditions (black) and in 100 nM Psora-4 (gray). Since some Kv1.3 channels are open at rest, Psora-4 depolarizes OBC in the rd1 retina (Figure 4A and Figure S8). Consequently, with Psora-4, the hyperpolarization amplitude (Figure 4B, CTR: −5.65 mV ± 0.74, N = 4; Psora-4: −11.37 mV ± 0.29, N = 3, ** *p* = 0.0019) and the response kinetics were significantly increased (Figure 4C, Tau on (s), CTR: 0.465 s ± 0.026, N = 4; Psora-4: 0.255 s ± 0.021, N = 3, ** *p* = 0.006) when the cell was driven towards the potassium equilibrium potential.

Since Kv1.3 immunolabeling was also upregulated in RGCs in *FVB rd1* retinas at *p210* and *p375* (Figure 1C and Figure S2), we also investigated the effect of Psora-4 on the activity of RGCs. For this, we used multi-electrode array (MEA) recordings with retinal whole mounts placed with the RGC-side facing toward the electrodes. Blue light stimulation (465 nm, 5 × 10^15^ photons/cm^2^/s) in the presence of Psora-4 resulted in more robust light responses (Figure 4D). Although the basal firing rate also increased during Psora-4 application (Figure 4E, N = 69 electrodes, **** *p* ≤ 0.0001), there was a significant increase in the Opto-mGluR6 mediated light-responses (Figure 4F, N = 69 electrodes, ** *p* = 0.002), increasing the signal-to-noise ratio of optogenetically elicited retinal output.

## 3. Discussion

The death of photoreceptors during degenerative retinal diseases, such as RP, ultimately leads to blindness [5,6,7,8]. OBCs, the first retinal interneurons downstream of the photoreceptors, were shown to survive for months to years after photoreceptor death [16,22,26], making them attractive targets for optogenetic vision restorative approaches [47]. Several optogenetic constructs have been introduced to OBCs to restore basic visual signaling [17,19]. The designer chimeric Opto-mGluR6 protein, consisting of the extracellular domains of melanopsin and the intracellular parts of mGluR6, restored bleach-resistant light responses with native mGluR6 kinetics operating at indoor light intensities when introduced into OBCs [20,22]. However, synaptic remodeling [26,28], hyperactivity [25], and changes in the expression and localization of proteins were observed in murine and human degenerating retinas [48,49,50]. It therefore remains unclear if such changes affect the quality of optogenetically restored vision. Specifically for the OBCs, it was described that they lose their dendrites [23,29,31] and, with that, downregulate big conductance potassium (BK) channels [31], which are normally expressed on the dendritic tips, playing a key role in keeping the OBCs in a “responsive” state [31].

Here, we describe that delayed rectifier potassium channels from the Kv1 family also express differently in the degenerated retina. While dendritic Kv1.1 and Kv1.2 channels are lost, Kv1.3 channel expression is triggered by degeneration, both in faster (*FVB rd1*) and slower (*C3H/HeOuJ rd1*) degenerating murine RP models [36]. Interestingly, Kv1.1 and Kv1.2 channels did not relocate to the OBC somas, such as described—for example—for the TRPM1 channel [51,52], but were lost and replaced by axonally expressed Kv1.3. We also found strong Kv1.3 expression in post-mortem ex vivo human retina cultures 12–48 h post-mortem; the isolated human tissue was already in an apoptotic state, probably a consequence of oxidative stress during the long transfer time from the patient to the laboratory. It was previously reported that apoptosis in cultured human retinas commences 12 h after retinal detachment [53]. We did not notice apoptosis in retinal cryosections from freshly processed wild-type mouse tissue (Supplementary Figure S4).

Kv1.3 channels are expressed in various cell types, including RGCs, where they play an important role in regulating the resting membrane potential and action potential firing [54,55]. In agreement with previous reports, we showed, in MEA recordings, that inhibition of the Kv1.3 hyperpolarizing activity in RGCs increased the basal firing rate. However, inhibition of Kv1.3 channels that are located in OBCs enhanced the signal-to-noise ratio of the RGC response, which could improve the quality of optogenetically restored vision.

In line with previous reports, we confirm that Kv1.1 and Kv1.2 dominate the outward currents in OBCs in the healthy retina [35]. In addition, we found that Kv1.3 currents dominate in OBCs of the degenerated retina. Kv1.1 is a low threshold channel activated by small increases in membrane potential [45], whereas Kv1.3 activates at more depolarized membrane potentials (−35 mV) [46]. The difference in Kv channel inventory in healthy versus degenerated OBCs is also apparent in the different inactivation kinetics in response to extreme depolarizing current steps (Supplementary Figure S7). At the relatively depolarized membrane potential of OBCs in the rd1 retina (Figure 3D,E,G), Kv1.3 is in a partially open state, and the background Kv1.3 conductance acts to reduce voltage responses of OBCs to current input (Figure 3I). Although Kv1.3 is pathologically overexpressed in OBCs of the rd1 retina, cells most certainly also express the Tandem Pore Domain potassium channels (leak potassium channels), which normally maintain the resting potential of cells. The fact that rd1 OBCs are still relatively depolarized (Figure 3D,E,G) suggests that there is also a large opposing depolarizing current. Given that there is no native mGluR6 input to the OBCs of the degenerated retina [20,24], a large part of this depolarizing current could be from open TRPM1 channels [56,57]. When Opto-mGluR6 is activated during light, TRPM1 channels will close to hyperpolarize the cell [20,22]. This hyperpolarization will cause open Kv1.3 channels to close. In addition, leak channels will drive a strong shift in membrane potential towards the potassium equilibrium potential. As a result, the amplitude and kinetics of Opto-mGluR6 triggered TRPM1 responses increase (Figure 4A,B and Figure S8).

Kv1.3 are highly expressed in microglia, where they were shown to contribute to microglial activation and neurotoxicity, both in the retina [40] and in the brain [58]. In the retina, Kv1.3 contributes to cell-autonomous death of RGCs by increasing the expression of proapoptotic genes [40]. Inhibition of Kv1.3 channels has been shown to have neuroprotective effects in animal models of neurodegenerative diseases. Selective Kv1.3 expression in the OBCs of the degenerating retina may therefore also hint towards a neurotoxic role.

In summary, our results open avenues for future combination optogenetic gene therapies with a pharmacological block of Kv1.3 channels to further improve the quality of restored vision. Advances in promoter and AAV design [29] render OBCs accessible to optogenetic gene therapy. Kv1.3 channels are already existing drug targets: antagonists are used in cancer treatments [59] and siRNA to reduce RGC cell death after optic nerve transection [40,41].

## 4. Materials and Methods

### 4.1. Animals

Males and females of *C57BL/6J_Opto-mGluR6* (*C57BL/6J*)*, FVB/NCrl_Opto-mGluR6* (*FVB rd1*), and *C3H/HeOuJ_Opto-mGluR6* (*C3H/HeOuJ rd1*) mice were used as previously described [31,36]. *FVB rd1* and *C3H/HeOuJ rd1* mice are characterized by a faster and slower progression of retinal degeneration, respectively [36,60]. These mouse lines are transgenic for the *Opto-mGluR6* optogene and the red fluorescent marker (TurboFP635) expressed selectively in their retinal OBCs [31,36].

### 4.2. Fluorescence-Activated Cell Sorting (FACS) and qPCR

*C57BL/6J* mice (p210) were used as control. *FVB/NCrl rd1* mice at p105, p210, and p375 were used as models of retinal degeneration at different time points [31,36]. Ten retinas from five mice were pooled together. After retinal dissociation, OBCs were sorted by TurboFP635 fluorescence with 39.57 ± 3.51% positive living cells extracted for *C57BL/6J*, 28.53 ± 3.24% for *FVB rd1* p105, 40.01 ± 7.34% for *FVB rd1* p210, and 25.79 ± 4.76% for *FVB rd1* p375. For *C3H/HeOuJ rd1* at p105 59.40 ± 11.38% at p210 29.44 ± 11.38% living cells were sorted. RNA was extracted and one-step quantitative reverse-transcription PCR (qPCR) was performed as previously described [31]. Values obtained for Kv1.3 channel RNA (F 5’-TCC GAA AAG CCC GGA GTA AC-3′, R 5’-CTG TGG AGT TGC CCG TTT TG-3’) were normalized against the RNA levels of the housekeeping gene RPL8, which was shown to remain stable during retinal degeneration [31,39].

### 4.3. Human Retinal Tissue and Culturing

All procedures were approved by the tenets of the Declaration of Helsinki and complied with governmental regulations. No ethics approval was required for this study as per national laws and regulations (Federal Act on Research involving Human Beings (Human Research Act, HRA 810.30, Art. 38)). The anonymized donor tissue was provided by the Department of Ophthalmology, Inselspital, Bern University Hospital, Bern, Switzerland. After receiving the eyes, the retina was extracted from the eyecup and placed in Ames’ medium containing gentamicin. Retinal explants were cut using scalpel blades from the mid-periphery of the retina, with a surface area of ca. 25 mm^2^. These pieces were then transported to tissue culturing wells and placed with RGCs facing upwards. Ames’ medium was removed from the wells and warmed, and the oxygenated culturing medium was added underneath the wells (ca. 1 mL). Empty wells contained 1 mL of distilled water. The medium exchange was performed every 48 h, where 500 µL of medium was discarded and replaced by 510 µL of fresh warm medium. The culturing medium consisted of DMEM/F-12 without L-Glutamine (BioConcept, Allschwill, Switzerland) supplemented with 0.1% Bovine serum albumin, 0.2 µM DL-Tocopherol, 1 mM Fumaric acid, 0.5 mM Galactose, 50 µg/mL Gentamicin, 1 mM Glucose, 0.5 mM Glycine, 10 mM HEPES, 0.02 µM Hydrocortisone, 1 µM Insulin, 25 µg/mL L-Alanyl-L-Glutamine, 50 µM Mannose, 10 µM *O*-acetyl-l-carnitine hydrochloride, 100 µL Penicillin-Streptomycin (100×), 0.02 µM Progesterone, 0.1 mM Putrescine dihydrochloride, 0.35 µM Retinol, 0.3 µM Retinyl acetate, 13 mM Sodium bicarbonate, 0.05 µM Sodium selenite, 0.003 µM 3,3′,5-Triiodo-l-thyronine sodium salt, 3 mM Taurine, and 0.5 mM ascorbic acid. The explants were cultured at 37 °C under 95% Air/5% CO_2_ atmosphere. Retinal tissue was cultured up to 10 days.

### 4.4. Immunohistochemistry

Immunolabeling of cryopreserved vertical retinal sections of *C57BL/6J* mice (p210) and *FVB rd1* mice (p210) and (p375), as well as human donors, was performed using standard protocols [31] with primary antibodies against Kv1.3 channels (1:100, Alomone Labs, Jerusalem, Israel, Cat# APC-002, RRID: AB_2040151), Kv1.1 (1:100, Abclonal Cat# A2992, RRID: AB_2764802), Kv1.2 (1:100, Abclonal Cat#A6295, RRID: AB_2766900), protein kinase C (1:750, Invitrogen, Carlsbad, CA, USA, Cat#sc8393, RRID: AB_628142), GαO (1:1000; Millipore, Darmstadt, Germany; Cat#mab3073, RRID: AB_94671), and secondary polyclonal antibodies against Alexa 488 (1:400, Invitrogen Cat# A11008, RRID: AB_143165 and Cat# A11006, RRID: AB_2534074) and CY3 (1:400, Invitrogen Cat#A10521, RRID: AB_2534030). Images were taken as a single optical section using a Zeiss LSM880 confocal microscope with a 40× objective (NA: 0.8). Increase in Kv1.3 axonal expression in OBCs was evaluated by colocalization analysis. Pearson’s coefficient was calculated with ImageJ-win32. Background in the red and green channels was subtracted, and JACop special plugin was used. Ten rod-OBCs axon terminals per mouse were analyzed and averaged for a total of thirty cells. Retinas were extracted from three *FVB rd1* p210 mice and three *C57BL/6J* p210. For Pearson’s coefficient analysis on the human retinal section for upregulation of Kv1.3 axonal expression, 43 rod-OBCs from 3 retinas of 3 human donors were analyzed. TUNEL assay was performed to apoptotic cells (In situ cell death Detection Kit, TMR red, Cat#: 12156792910, Sigma, Buchs, Switzerland). DAPI was used as nuclear counterstain and anti-Gαo and PKCα as OBC labels. TUNEL+ OBCs were plotted against the total number of OBCs counted in an area of 27.03 ± 2.15 μm.

### 4.5. Patch-Clamp Recordings

#### 4.5.1. Solutions and Drugs

For whole-cell, perforated patch recordings and for retinal dissections, sodium hydrogen carbonate (NaHCO_3_) buffered Ames’ medium was used [31,61]. The pipette solution contained 110 mM potassium chloride (KCl), 10 mM sodium chloride (NaCl), 10 mM HEPES, 1 mM guanosine triphosphate (GTP), 1 mM adenosine triphosphate (ATP), 0.1 mM cyclic guanosine monophosphate (cGMP), 0.01 mM cyclic adenosine monophosphate (cAMP), 5 mM ethylene glycol-bis(2-aminoethylether)-*N*,*N*,*N*′,*N*′-tetraacetic acid (EGTA), 1 mM magnesium chloride (MgCl_2_), and 0.5 mM calcium chloride (Cl_2_, pH 7.4) [31]. In the perforated patch experiment, a saturated solution of Amphotericin B (Sigma Aldrich) was obtained in DMSO and then dissolved to a final concentration of 1:200 in the intracellular solution [22]. Psora-4 (Tocris) was used as a potent and specific Kv 1.3 channel blocker and TEA as an unspecific potassium channel blocker. Stock solutions of Psora-4 were prepared in dimethyl sulfoxide (DMSO) and stored at −20 °C and then dissolved in Ames’ medium to the working concentration on the day of the experiment. TEA was directly dissolved at the working concentration in the Ames’ medium on the day of the experiment. Wash-in was reached in 2 min. The Wash-in and wash-out timeline was applied as previously described [31]. The calculated liquid junction potential of 7.5 mV was subtracted from all traces.

#### 4.5.2. OBC Identification

Mice were euthanized using cervical dislocation and retinas were rapidly removed. A total of 5 *C57BL/6J* mice and 5 *FVB rd1* (p210) were used for the patch-clamp recordings. Retina slices of *C57BL/6J* mice and whole mounts of *FVB rd1* (p210) were obtained as previously described [22,31,42]. The lack of photoreceptors in the FVB *rd1* retina allows direct access to the OBCs in the retina whole mount, and no difference has been found in the electrophysiological properties of bipolar cells in the two preparations as previously described [31]. OBC somas were identified via Turbo FP635 fluorescence with an upright microscope (Nikon Eclipse E600FN, Tokyo, Japan) equipped with an infrared GP-CAM3 Altair Astro camera [31]. To visualize recorded cells, 0.03% Lucifer yellow potassium salt (Sigma Aldrich) was added to the intracellular solution [31].

#### 4.5.3. Voltage and Current Clamp Recordings

Patch-clamp recordings were performed with an Axopatch 200B amplifier (Axon instruments) in the whole cell configuration. Retinal slices and whole mounts were perfused at a flow rate of 5 mL/min with gassed Ames’ medium (95% O_2_ and 5% CO_2_) as previously described [31]. Patch pipettes (Harvard apparatus) were pulled at a resistance of 8–12 MΩ using a ZEIT DMZ puller (Martinsried, Germany). Whole-cell voltage steps protocols (from −80 mV to +100 mV, Δ = 10 mV) were used to elicit the Kv 1.3 current, and current steps protocols (−10 pA to +70 pA, Δ = 10 pA) were applied to evaluate the effect of the Kv 1.3 block with Psora-4 on the cell membrane potential.

#### 4.5.4. Light Responses from OBCs in the *FVB rd1* Retina

*FVB rd1* p210 were used. Since *FVB rd1* mice lack the photoreceptor layer, patching was performed in whole-mount retinas attached to a coverslip as previously described [22,31,42]. Perforated patch configuration with Amphotericin B was used. The 1-s-long blue (465 nm) light stimulus was generated with a pE-4000 epi-fluorescence light source from CoolLED (CoolLED, Andover, UK). The light intensity was kept to 5 × 10^15^ photons/cm^2^/s. Since voltage recordings in response to light stimulation from OBCs were technical, and recordings were often short-lived, we recorded from one group of control cells and then from a second group of cells which contained Porsa-4 in the bath solution (Figure 4A–C).

### 4.6. Multi Electrode Array Recordings

Experiments were performed on healthy *C57BL/6J* (N = 2 animals, 4 retinal explants) and *FVB rd1* (N = 3 animals, 8 retinal explants) aged to p210. Mice were dark-adapted for 60 min and subsequently sacrificed using isoflurane and cervical dislocation. Following enucleation, eyes were dissected under dim red-light conditions in an Ames’ medium (Sigma-Aldrich) that had been oxygenated for at least 60 min prior to the procedure with carbogen (95% O_2_/5% CO_2_). The retinas were placed on multi-electrode arrays (MEAs; 60MEA200/30iR-Ti; (Multi Channel Systems MCS GmbH, Reutlingen, Germany) coated with Corning™ Cell-Tak Cell and Tissue Adhesive (Corning, New York, NY, USA), with the ganglion cells facing the electrodes. The MEA was placed into the MEA recording device (MEA2100-System; Multi Channel Systems MCS GmbH) positioned on a stage of a Zeiss Axioskop. Tissue was perfused with gassed, bicarbonate buffered Ames’ medium (34 °C; 5 mL/min; Merck, Darmstadt, Germany) for 30 min before the start of the recording protocol. Light stimulation was delivered via the 5× objective positioned above the MEA recording device with the pE2 light stimulator used as a light source (precisExcite, CoolLED, Andover, UK). Light pulses were triggered using a TTL signal generator (STG2008, Multi Channel Systems MCS GmbH). The intensity and duration of light stimulation was 465 nm, 5 × 10^14^ photons/cm^−2^/s, unless stated otherwise. The light stimulation protocol consisted of 3 consecutive light flashes (1 s every 120 s), followed by bath application of Psora-4 in Ames’ medium (100 nM, 5 min). Subsequently, the light stimulation protocol was repeated (with Psora-4 still present in the perfusion). Recorded signals were collected, amplified, and digitized at 25 kHz using MCRack software (version 4.6.2, Multi Channel Systems MCS GmbH Reutlingen, Germany). The signals were filtered using a 2nd order Butterworth high-pass filter (cut-off frequency = 200 Hz), and multi-unit spike activity was defined as an electrical activity below 3.5–5 SDs of baseline activity and set specifically for each recording electrode based on the baseline noise at the beginning of the recording. Multi-unit spike-time occurrences were extracted and analyzed in Matlab (version R2021b, MathWorks, Natick, MA, USA). Three light flashes before and after the application of Psora-4 were averaged into a single trace. Electrodes were deemed light-responsive if at least 1-time bin during or after the light flash crossed a threshold defined as averaged baseline activity before the light stimulation + 3 SDs. In the case of cells that were silent prior to light stimulation, a threshold of 40 Hz was used. Only cells that passed these filtering thresholds before as well as after the bath application of Psora-4 were used for further analysis. The basal firing rate, as well as the light-evoked firing rate (defined as peak light-evoked spiking—basal activity), were extracted and, subsequently, statistical analysis was performed in GraphPad (Prism, version 9.3.1) using the Wilcoxon matched-pairs signed rank test. Normal distribution was rejected in all the datasets using both the Shapiro–Wilk and Kolmogorov–Smirnov tests.

### 4.7. Data Analysis and Statistics

Electrophysiological data were analyzed with pCLAMP 10.7 (Molecular Devices, San Jose, CA, USA) and Prism 5 (GraphPad Software, San Diego, CA, USA). We used the parametric Student’s t-test to compare data coming from cells tested in CTR condition and, consequently, with the drug application and the non-parametric Mann–Whitney test for cells tested independently in the mentioned conditions. In the figures (*) indicates *p* ≤ 0.05, (**) indicates *p* ≤ 0.02, (***) indicates *p* ≤ 0.001, and (****) indicates *p* ≤ 0.0001. Results are presented as average values ± standard error (SEM). To calculate the reversal membrane potential (Erev), I/V curves were fit with a 7 variables polynomial equation **(*y = ax*^6^ + *bx*^5^ + *cx*^4^ + *dx*^3^ + *ex*^2^ + *f* + *g*)**. Wolfram Alpha software (https://www.wolframalpha.com/input/?i=software) was used to solve the polynomial equation and calculate Erev. The data from the acquired FACS files were analyzed as previously described [31].

## Data Availability

The datasets used and/or analyzed during the current study are available from the corresponding author upon reasonable request.

## Supplementary Materials

The following supporting information can be downloaded at: https://www.mdpi.com/article/10.3390/ijms241814207/s1.
