# Supplementary material for: Selective Block of Upregulated Kv1.3 Potassium Channels in ON-Bipolar Cells of the Blind Retina Enhances Optogenetically Restored Signaling
Source: Int J Mol Sci. 2023 Sep 18;24(18):14207. doi: 10.3390/ijms241814207 (PMC10531754; doi:10.3390/ijms241814207)
Supplement: Supplementary file 1 [file ijms-24-14207-s001.zip › ijms-2418444-supplementary.pdf]

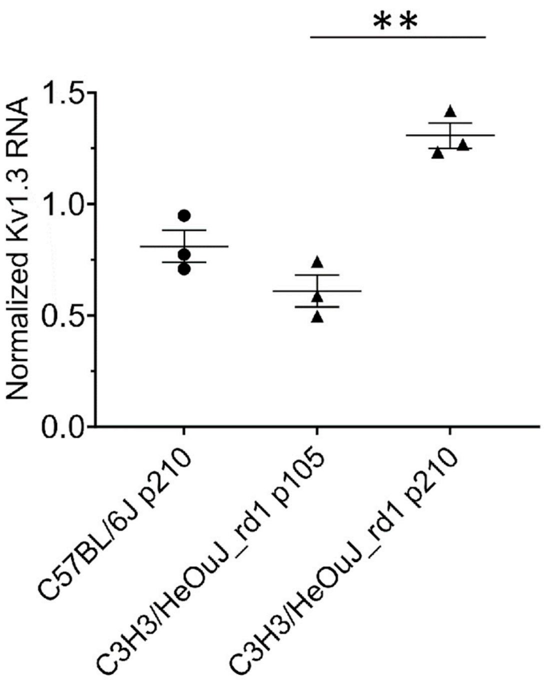

Figure S1. Kv1.3 expression increases during degeneration in *C3H3/HeOuJ rd1* mouse model. Kv1.3 channel RNA levels in OBCs from *C57BL/6J* mice (p 210) and *C3H3/HeOuJ rd1* mice (p105) (p210), **p=0.015.

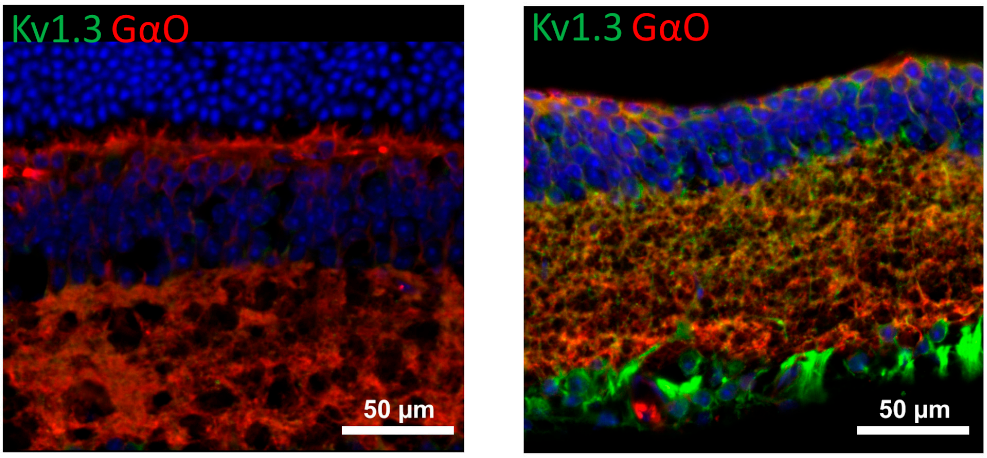

Figure S2. Kv1.3 expression increases during degeneration in OBCs. Anti-Kv1.3 immunolabeling (green) on retinal cryosection of *C57BL/6J* mice at p210 (left), *FVB rd1* mice at p210 (right). OBCs were identified by anti-GαO immunolabeling (red). Images were taken as single optical sections (770 nm) on a Zeiss LSM880 confocal microscope (40×, NA: 1.3).

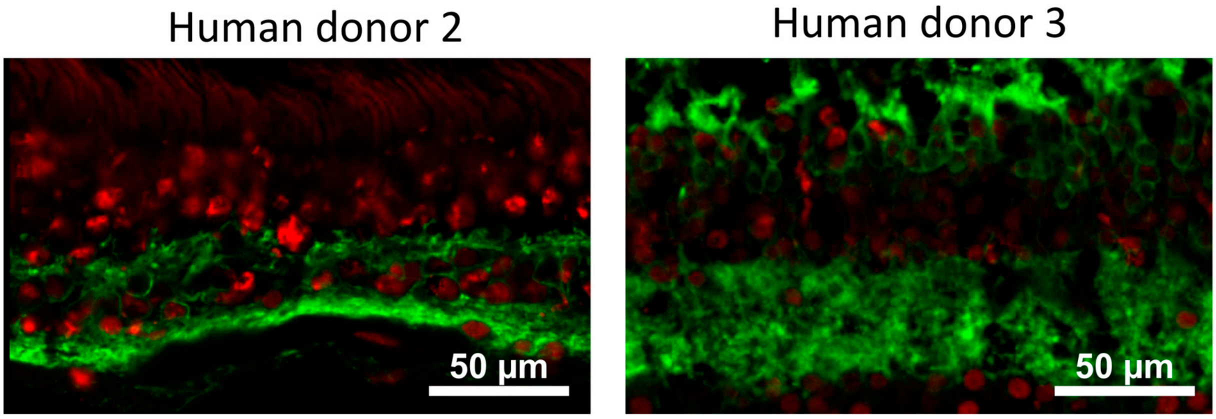

Figure S3. TUNEL+ (red) OBCs (green) in the human retina at 0 days of culture from 2 different donors (donor 2 left, donor 3 right). OBCs were stained with anti-GαO immunolabeling. Images were taken as single optical sections (770 nm) on a Zeiss LSM880 confocal microscope (40×, NA: 1.3).

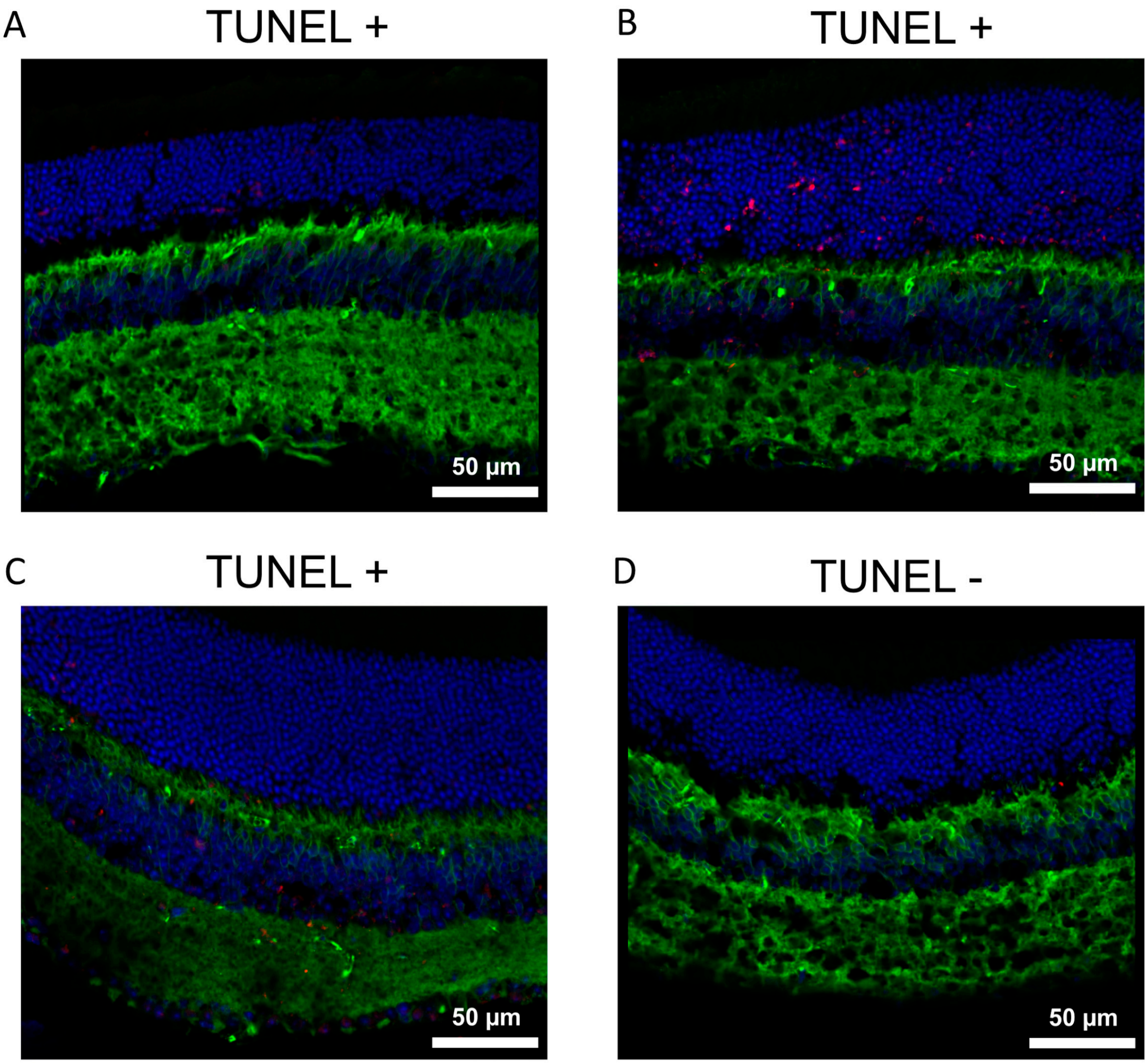

Figure S4. (A)(B)(C) TUNEL+ (red) OBCs (green) in *C57BL/6J* retina slice from 3 biological samples; (D) TUNEL - staining as a negative control on *C57BL/6J* retina slice. OBCs were stained with anti-GαO immunolabeling. Images were taken as single optical sections (770 nm) on a Zeiss LSM880 confocal microscope (40×, NA: 1.3).

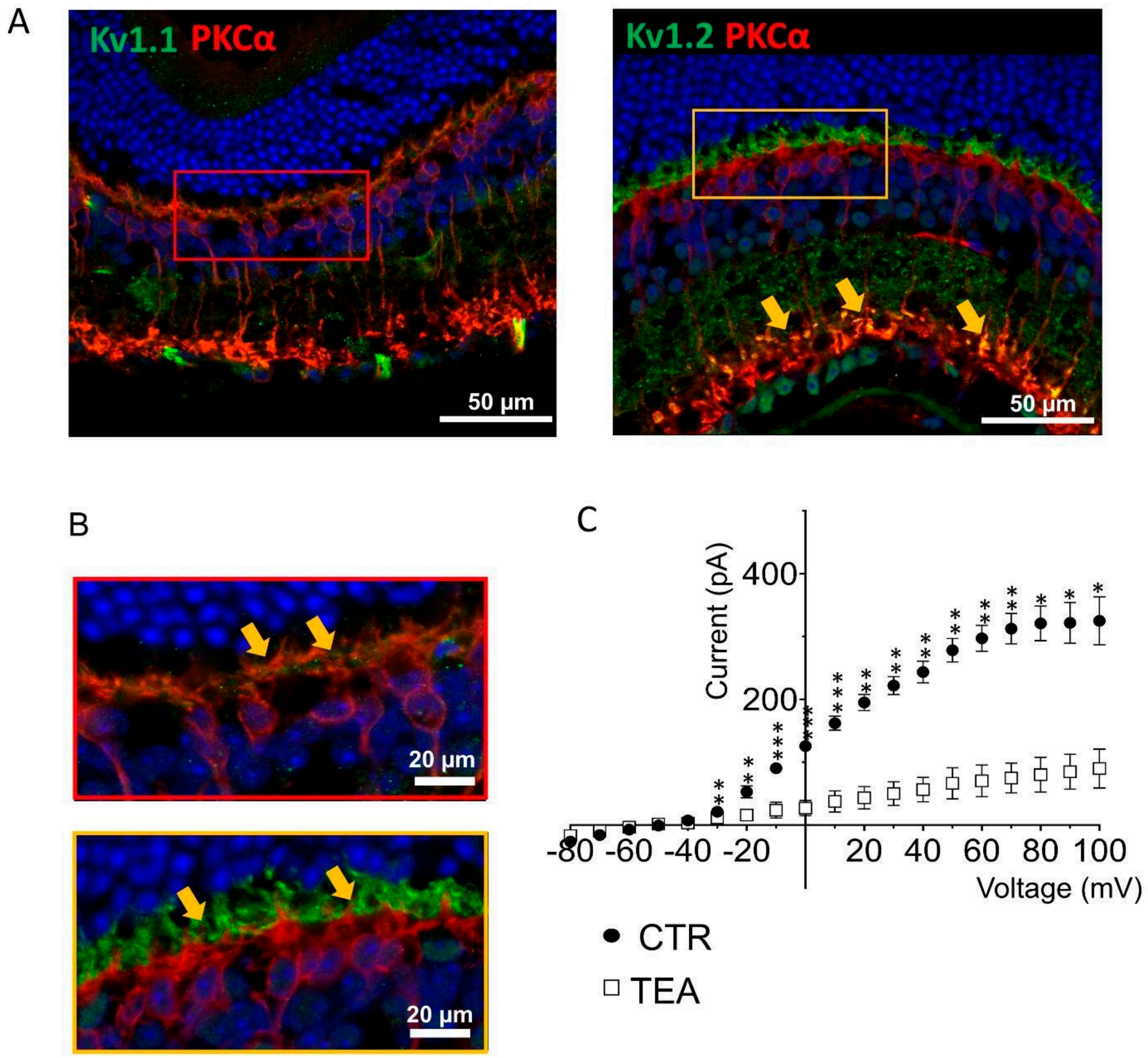

Figure S5 Kv1.1 and Kv1.2 are localized in the OBCs dendrites and axon terminals in the healthy mouse retina. (A) anti-Kv1.1 immunolabeling (green) (left) and anti-Kv1.2 (right) on retinal cryosection of *C57BL/6* mice at p210. OBCs were identified by anti-PKC*α* immunolabeling (red). Images were taken as single optical sections (770 nm) on a Zeiss LSM880 confocal microscope (40×, NA: 1.3); (B) Magnification of figure A with Kv1.1 (top) and Kv1.2 (bottom); Kv1.1 is expressed in OBC dendrites and Kv1.2 in OBC dendrites and axon terminals. (C) I/V relationship (step protocol from -80 mV to 100 mV, Δ = 10 mV) in control condition (CTR, n= 3) and with the bath application of the potassium channel blocker 10 mM TEA (n=3) (-30 mV, **p=0.0144; -20 mV, **p=0.0037 ; -10 mV ***p=0.00033 ; 0mV ***p=0.00075 ; 10mV ***p=0.0030 ; 20mV, **p=0.0037 ; 30mV, **p=0.0066 ; 40mV, **p=0.0094 ; 50mV, **p=0.0091 ; 60mV, **p=0.0089; 70mV, **p=0.0135 ; 80mV, *p=0.0210 ; 90mV, *p=0.0267 ; 100mV, *p=0.0375);

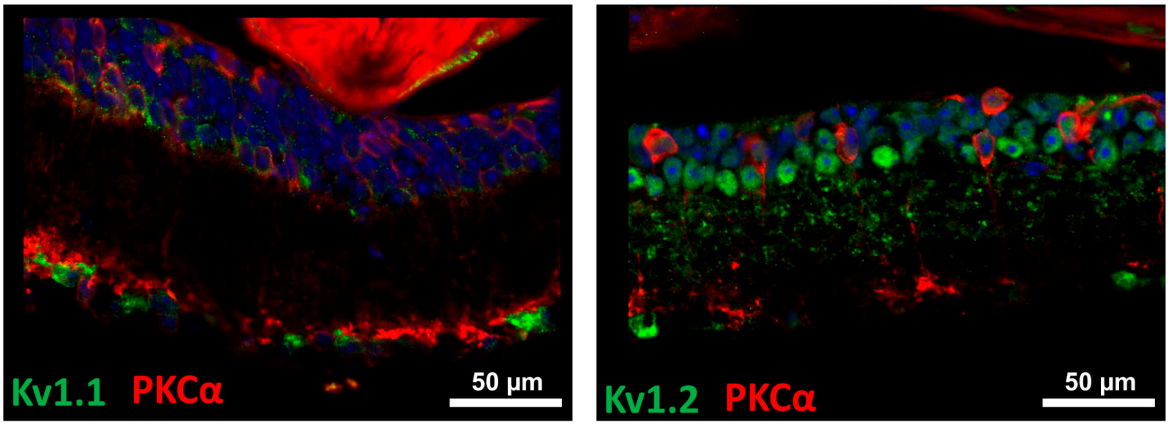

Figure S6. Kv1.1 and Kv1.2 channels expression in OBCs in *FVB rd1* retina. anti-Kv1.1 (left) and anti-Kv1.2 (right) immunolabeling (green) on retinal cryosection of *FVB rd1* mice at p210 (left). ON-RBCs were identified by anti-PKCα immunolabeling (red). Images were taken as single optical sections (770 nm) on a Zeiss LSM880 confocal microscope (40×, NA: 1.3).

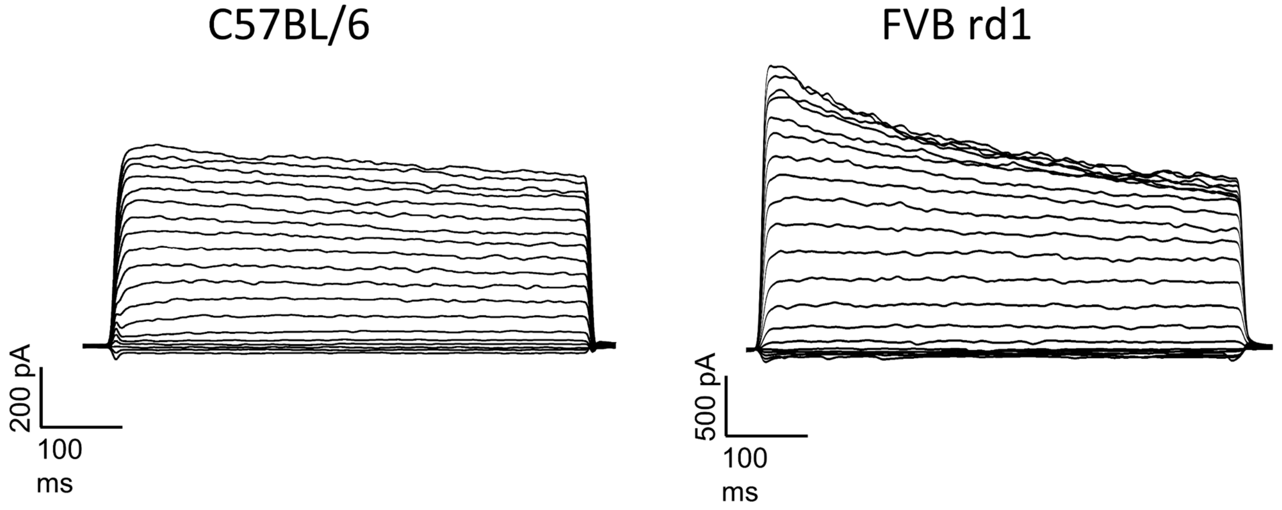

Figure S7. Example traces of voltage step protocol (-80 mV to 100 mV, Δ=10 mV) for a C57BL/6J (left) and for an FVB rd1 (right) ON-bipolar cell. The off-kinetics are clearly different in the two traces, with a Tau(off) of 564.37 ± 147.72 ms in C57BL/6 ON-bipolar cells and a Tau(off) of 43.42± 22.43 ms in FVB rd1 ON-bipolar cells, in line with a change from expressing Kv1.1/1.2 channels in the healthy retina to expressing predominantly Kv1.3 in the degenerating rd1 retina.

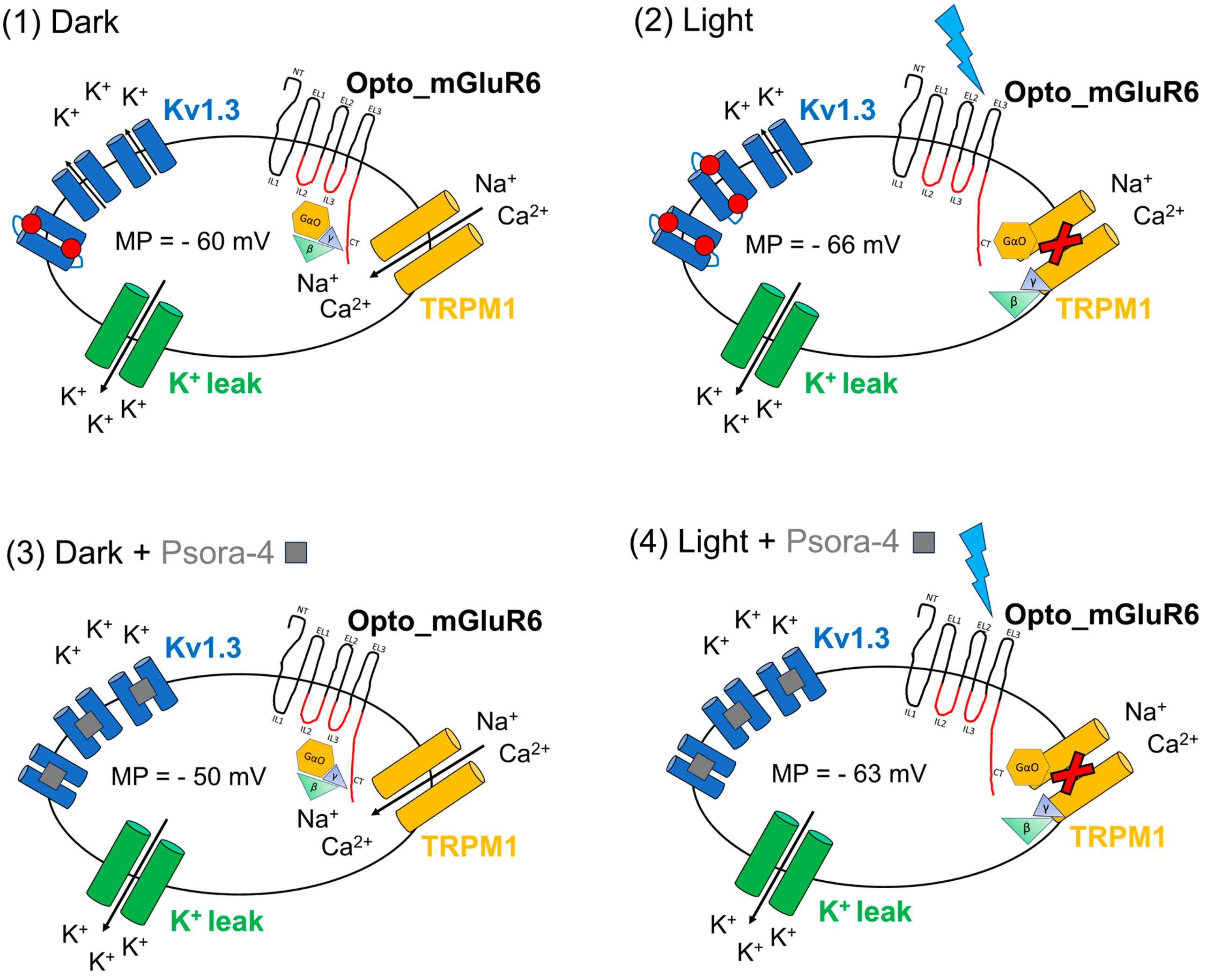

Figure S8. Cartoon depicting the potential underlying mechanism of membrane potential tuning in OBCs during optogenetic activation via Opto-mGluR6 with and without Psora-4 inhibition of Kv1.3 channels, shown in Fig. 4A. (1) In the dark, Opto-mGluR6 is inactive, TRPM1 open and depolarizing the OBC, while the majority of Kv1.3 channels is open and leak potassium channels (K+ leak) are hyperpolarizing. (2) Light activates Opto-mGluR6, TRPM1 closes, Kv1.3 becomes partially inactive due to hyperpolarization driven by TRPM1, leak potassium current drives membrane potential towards the potassium equilibrium potential. (3) As (1), but Psora-4 inhibits hyperpolarizing Kv1.3 channels, which depolarizes the membrane potential. (4) As (2), but now all Kv1.3 channels are blocked from the beginning by Psora-4, therefore the membrane potential may be slightly depolarized as opposed to (2). The main hyperpolarization driver is the closure of TRPM1. Note: The given membrane potentials are actual experimental average values. There may be, however, some clamping issues due to the very thin processes and high access resistances of OBCs, potentially causing a slight artificial shift in the K reversal potential.
